# Milrinone Attenuates Arteriolar Vasoconstriction and Capillary Perfusion Deficits on Endotoxemic Hamsters

**DOI:** 10.1371/journal.pone.0117004

**Published:** 2015-02-03

**Authors:** Marcos Lopes de Miranda, Sandra J. Pereira, Ana O. M. T. Santos, Nivaldo R. Villela, Luiz Guilherme Kraemer-Aguiar, Eliete Bouskela

**Affiliations:** 1 Department of Internal Medicine, Division of Critical Care, Faculty of Medical Sciences, Rio de Janeiro State University, Rio de Janeiro, RJ, Brazil; 2 Pediatric Cardiac Intensive Care Unit, Perinatal Barra, Rio de Janeiro, RJ, Brazil; 3 Institute Fernandes Figueira, Oswaldo Cruz Foundation—FIOCRUZ, Rio de Janeiro, RJ, Brazil; 4 Department of Surgery, Division of Anesthesiology, Faculty of Medical Sciences, Rio de Janeiro State University, Rio de Janeiro, RJ, Brazil; 5 Department of Internal Medicine, Division of Endocrinology, Faculty of Medical Sciences, Rio de Janeiro State University, Rio de Janeiro, RJ, Brazil; 6 Laboratory for Clinical and Experimental Research in Vascular Biology—BioVasc, Biomedical Center, Rio de Janeiro State University, Rio de Janeiro, RJ, Brazil; Indian Institute of Science, INDIA

## Abstract

**Background and Objective:**

Apart from its inotropic property, milrinone has vasodilator, anti-inflammatory and antithrombotic effects that could assist in the reversal of septic microcirculatory changes. This paper investigates the effects of milrinone on endotoxemia-related microcirculatory changes and compares them to those observed with the use of norepinephrine.

**Materials and Methods:**

After skinfold chamber implantation procedures and endotoxemia induction by intravenous Escherichia coli lipopolysaccharide administration (2 mg.kg-1), male golden Syrian hamsters were treated with two regimens of intravenous milrinone (0.25 or 0.5 μg.kg-1.min-1). Intravital microscopy of skinfold chamber preparations allowed quantitative analysis of microvascular variables. Macro-hemodynamic, biochemical, and hematological parameters and survival rate were also analyzed. Endotoxemic non-treated animals, endotoxemic animals treated with norepinephrine (0.2 μg.kg-1.min-1), and non-endotoxemic hamsters served as controls.

**Results:**

Milrinone (0.5 μg.kg-1.min-1) was effective in reducing lipopolysaccharide-induced arteriolar vasoconstriction, capillary perfusion deficits, and inflammatory response, and in increasing survival. Norepinephrine treated animals showed the best mean arterial pressure levels but the worst functional capillary density values among all endotoxemic groups.

**Conclusion:**

Our data suggests that milrinone yielded protective effects on endotoxemic animals’ microcirculation, showed anti-inflammatory properties, and improved survival. Norepinephrine did not recruit the microcirculation nor demonstrated anti-inflammatory effects.

## Introduction

Sepsis is an infection-related systemic inflammatory syndrome with high incidence, morbidity, mortality, and cost to healthcare system [1, 2]. In sepsis syndrome, the inflammatory response is associated with microthrombosis and microvascular vasoconstriction, leading to microcirculatory dysfunction, the critical first stage in sepsis progression towards tissue hypoxia, organ failure, and death [3, 4]. Impaired microcirculatory perfusion occurs in patients with septic shock despite restoration of intravascular volume and/or normalization of blood pressure [5]. Thus, drugs that assist in microcirculatory opening could be decisive for sepsis treatment [3].

Short-acting vasodilators have been used to recruit the microcirculation in patients with septic shock, mainly pediatric ones who remain in a state of low cardiac output and high systemic vascular resistance despite adequate therapy with an inotropic agent [5–7]. Although the effects of vasodilators on the improvement of microvascular function are well-established, they are rarely used in septic adults because in this population a hemodynamic state with high systemic vascular resistance is quite uncommon and administration of such drugs may result in profound hypotension [8]. An alternative approach to open the microcirculation is based on the use of type III phosphodiesterase (PDE-3) inhibitors, like the inodilator milrinone. Theoretically, with this class of drugs it would be possible to achieve microvessel dilatation while maintaining satisfactory perfusion pressure by increased cardiac output. PDE-3 inhibitors have been used in pediatric patients with sepsis of different etiologies with promising results [9, 10]. Furthermore, milrinone has already been associated with improved cardiac output and tissue perfusion in experimental sepsis, as evidenced by measurements of central venous saturation and blood lactate concentration [11]. Besides its hemodynamic effects, there is increasing evidence that milrinone is able to interfere with the inflammatory cascade and platelet aggregation [12–14]. Together, these effects may contribute to restoration of microcirculatory function, improving tissue perfusion and reducing organ failure

Based on these findings, we hypothesized that milrinone infusion could attenuate microcirculatory derangements evoked by sepsis, even during the hyperdynamic phase, but this subject has been poorly explored in the literature. Thus, the present controlled experimental study was carried out to investigate the microcirculatory effects of milrinone on an endotoxemia rodent model that allows *in vivo* studies of the microcirculation and to compare its effects with those observed with norepinephrine (an extensively studied and commonly used drug).

## Materials and Methods

Experiments were performed on 50 male golden Syrian hamsters (*Mesocricetus auratus*, ANILAB, Animais de Laboratório, Paulínia, SP, Brazil) weighing between 60 and 90 g. Animals were housed, one per cage, under controlled conditions of light (12:12 hours light/dark cycle) and temperature (21.0±1.0°C), with free access to water and standard chow (NUVILAB CR1, Quimtia S/A, Colombo, PR, Brazil). All procedures were approved by the Rio de Janeiro State University Animal Care and Use Committee, Rio de Janeiro, RJ, Brazil (protocol number CEUA/060/2010) and are consistent with the United States National Institutes of Health Guide for the Care and Use of Laboratory Animals (National Research Council, Update 2011).

### Animal preparation

The chamber implantation procedure has been described previously by Endrich et al. [15] in details. Briefly, under anesthesia with sodium pentobarbital (90 mg.kg^-1^ intraperitoneal injection; Hypnol 3%, Syntec, Cotia, SP, Brazil) animals’ dorsal hair was shaved and depilated with a commercial hair-removing solution. After that, the dorsal skin of the back was lifted away from the animal, creating a skinfold. Then, this skinfold was sandwiched between two titanium frames and one of its layers was microsurgically excised in a circular area of 15 mm in diameter. The remaining layer, consisting of epidermis, subcutaneous tissue, and thin striated skin muscle (*panniculus carnosus* muscle) was covered with a removable circular cover glass incorporated into one of the metal frames, creating the window chamber. After a recovery period of 6 days, animals were re-anesthetized and the left carotid artery catheterized (polyethylene-50 catheter) allowing continuous hemodynamic monitoring and blood sampling. The left jugular vein was also catheterized (polyethylene-10 catheter) for fluid infusion and drug injection. These catheters were tunneled under the skin, exteriorized at the dorsal side of the neck, filled with heparinized saline solution (40 IU.ml^-1^), and attached to the chamber frame with tape. Experiments were performed on awake animals after 24 hours of catheter implantation.

### Hemodynamic monitoring

Mean arterial blood pressure (MAP) was continuously monitored during the experimental period through the arterial catheter and a pressure transducer. Analog pressure signals were digitized (MP100 Data Acquisition System, BIOPAC Systems, Goleta, CA, USA) and processed using data acquisition software for hemodynamic experiments (AcqKnowledge Software v. 3.5.7, BIOPAC Systems, Goleta, CA, USA). Heart rate (HR) was determined from the pressure trace and expressed as beats per minute (bpm).

### Intravital microscopy

The unanesthetized animal was placed in a restraining plexiglass tube attached to the stage of an intravital microscope (Ortholux, Leitz, Wetzlar, Germany) equipped with an epifluorescence assembly (100-W HBO mercury lamp with filter blocks, Leitz, Wetzlar, Germany). The body temperature of the hamsters was maintained with a heating pad placed near the animal controlled by a rectal thermistor (LB750, Uppsala Processdata AB, Uppsala, Sweden). Moving images of the microcirculation were obtained using a 20x objective (CF SLWD Plan EPI 20x/0.35 Achromat Objective WD 20.5 mm, Nikon, Tokyo, Japan) and a charge-coupled device digital video camera system (SBC-320P B/W Camera, Samsung, Seoul, South Korea) resulting in a total magnification of 800-fold at the video monitor. Microcirculatory acquired images were recorded as video files in digital media for later evaluation. Quantitative off-line analysis of videos was performed using Cap-Image 7.2, a computer-assisted image analysis system (Dr.Zeintl Biomedical Engineering, Heidelberg, Germany;[16]) by an investigator blinded to drug treatment. In each animal, 2 arterioles, 2 venules, and 10 capillary fields were chosen taking into account the absence of inflammation or bleeding in the microscopic field and the presence of histological landmarks that could facilitate the subsequent return to the same field since the same vessels and capillary fields were studied throughout the experiment. Arteriolar and venular mean internal diameters were measured as perpendicular distance (in micrometers) between the vessel walls. Functional capillary density (FCD) was considered to be the total length (in centimeters) of spontaneously red blood cell (RBC)-perfused capillaries per square centimeter of tissue surface area (cm.cm^-2^). RBC velocity in capillaries (RBC-Vel) was assessed by frame-to-frame analysis and determined as the ratio between the capillary distance traveled by an erythrocyte and the time required for this displacement (expressed as mm.s^-1^). One capillary per capillary field was studied in each animal during RBC-Vel assessment. The selection of this capillary was based on two criteria: it should be representative of the mean RBC-Vel of that field and have good image quality for reliable analysis.

### Biochemical and hematological parameters

Blood samples were withdrawn from the arterial catheter and immediately analyzed in a point-of-care lactate and blood gas analyzer (i-STAT System/CG4+ cartridge, Abbott Laboratories, Abbott Park, IL, USA) for arterial lactate concentrations, pH, and bicarbonate level (HCO_3_). Arterial blood glucose was also immediately measured with a glucometer (OneTouch Ultra, Lifescan, Johnson & Johnson, Milpitas, CA, USA). Hematocrit was determined by the microhematocrit method using arterial blood samples collected into heparinized capillary tubes. Arterial blood leukocytes were manually counted by standard laboratory method. Briefly, after leukocytes staining with gentian violet and erythrocytes hemolysis by acetic acid (Türk’s solution, Sigma-Aldrich, St. Louis, MO, USA), leukocytes were counted in a defined volume using a Neubauer counting chamber and an optical microscope equipped with a 10x/0.25 objective (BIOVIDEO, BEL Photonics, Monza, Italy). Leukometry was expressed as the number of leukocytes per mm^3^.

### Experimental protocol

Animals were suitable for experiments if their baseline hemodynamic variables were within the normal range. Animals with signs of inflammation and/or bleeding in the chamber were excluded from the study. Included hamsters were randomly allocated to one of five study groups: (1) Control (n = 10)—non-endotoxemic animals; (2) LPS (n = 10)—animals without further treatment after endotoxemia induction; (3) Milrinone 25 (n = 10)—one hour after endotoxemia induction, animals were treated with a bolus dose of milrinone (25 μg.kg^-1^; 0.07 ml) followed by a continuous intravenous (IV) infusion (0.25 μg.kg^-1^.min^-1^) maintained at a 0.1 ml.h^-1^ infusion rate for 5 hours; (4) Milrinone 50 (n = 10)—one hour after endotoxemia induction, animals were treated with a bolus dose of milrinone (50 μg.kg^-1^; 0.07 ml) followed by a continuous IV infusion (0.50 μg.kg^-1^.min^-1^) maintained at a 0.1 ml.h^-1^ infusion rate for 5 hours; (5) Norepinephrine (n = 10)—one hour after endotoxemia induction, a continuous IV infusion of norepinephrine (0.2 μg.kg^-1^.min^-1^) was initiated and maintained at a 0.1 ml.h^-1^ infusion rate for 5 hours. Fresh solutions of milrinone and norepinephrine were prepared at the time of each experiment by dilution in normal saline.

At the beginning of the experiment, animals were given 30 minutes to adapt to restraining plexiglass tube before baseline variables were measured. Immediately after baseline determination of hemodynamic and microcirculatory parameters, hamsters belonging to Control group (non-endotoxemic animals) received an IV injection of 0.2 ml of normal saline, while endotoxemia was induced in all other groups by an IV injection of 2 mg.kg^-1^ of *Escherichia coli* serotype 055:B5 lipopolysaccharide (LPS; Sigma-Aldrich, St. Louis, MO, USA) diluted in normal saline (total volume of 0.2 ml).

**Fig 1:**
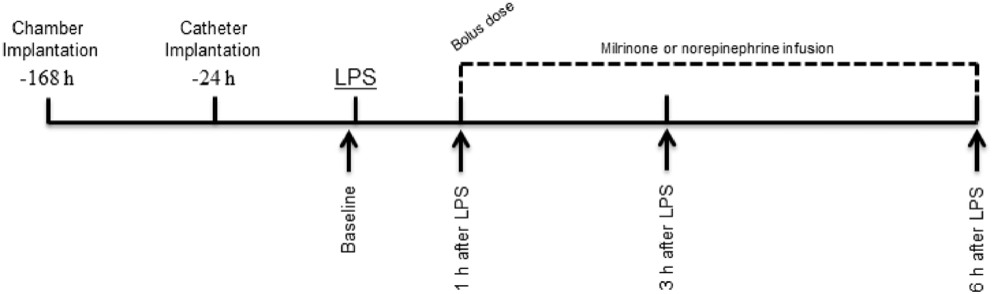
Schematic representation of the experimental protocol. After baseline determination of hemodynamic and microcirculatory parameters (Baseline) LPS was administrated. Sequential measurements were performed after one, three, and six hours of LPS injection (arrows). Blood sampling for biochemical and hematological analysis was performed in all groups at the end of the study period (6 h after LPS).

As shown in Fig. 1, sequential measurements of hemodynamic and microcirculatory parameters were performed at four time points: at baseline and after 1, 3, and 6 hours of LPS injection. At the second time point (1 h after LPS), measurements were performed immediately before drug administration. Blood sampling for biochemical and hematological analysis was performed in all groups at the end of the study period (6 hours after LPS injection).

### Survival analysis

After the intravital microscopy phase of the experiments, animals were returned to their individual cage in the *vivarium* with free access to water and standard chow and monitored for survival, pain and/or distress three times per day for 7 days.

In order to minimize suffering of the animals during survival analysis, animals were humanely euthanized by an intravenous overdose of sodium pentobarbital (>200 mg.kg^-1^) when serious impairment of ambulation (making animals unable to reach food or water easily), inability to remain upright, lethargy or persistent recumbency, lack of responsiveness to manual stimulation, evidence of muscle atrophy or other signs of emaciation, bleeding, seizures, breathing difficulty, prolonged palpable hypothermia, or any other sign of pain and/or distress was observed. After 7 days, surviving animals were euthanized by a lethal dose of sodium pentobarbital.

### Statistical analysis

Results are expressed as means ± standard deviation of the mean (SD) for each group, unless otherwise noted. Sample size was based on previous experience with the endotoxemia and microcirculation models used. Normally, with these models it is possible to observe significant differences between groups with the inclusion of 5–6 animals per group. In this study, a larger number of animals (10 in each group) was used because of the lack of data regarding the use of milrinone in association with the hamster skinfold window chamber model. FCD and RBC-Vel data are presented as ratios relative to baseline values. All hemodynamic and microcirculatory measurements were compared with baseline of the same group and between groups at the same time point. Statistical differences within and between groups were determined by Friedman and Kruskal-Wallis tests, followed, when appropriate, by Dunn’s multiple-comparisons test for *post hoc* analysis. Survival curves were obtained using Kaplan-Meier procedure and Mantel-Cox log-rank test was applied for determination of significant differences between study groups. In Milrinone 50 and Norepinephrine groups, FCD was correlated with MAP using Spearman rank correlation. All statistical analyses were performed using GraphPad Prism 6.03 (GraphPad Software, La Jolla, CA, USA) and the significance level was set as *p* <0.05.

## Results

The average body weight of hamsters was 84.4±4.2 g with no significant differences among groups. All animals survived the intravital microscopy phase of the experimental protocol and underwent survival analysis leaving no missing data for statistical analysis.

### Hemodynamic alterations

**Fig 2:**
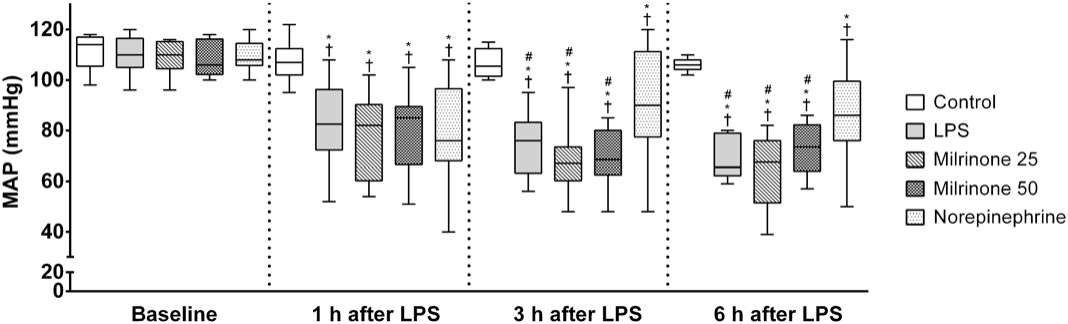
Evolution of mean arterial pressure during the experimental period. Sequential measurements were performed after one, three, and six hours of LPS injection. Control, non-endotoxemic animals (n = 10); LPS, endotoxemic animals (n = 10); Milrinone 25, endotoxemic and milrinone 0.25 μg.kg^-1^.min^-1^ treated animals (n = 10); Milrinone 50, endotoxemic and milrinone 0.50 μg.kg^-1^.min^-1^ treated animals (n = 10); Norepinephrine, endotoxemic and norepinephrine treated animals (n = 10). † *p* <0.05 *vs*. group baseline; * *p* <0.05 *vs*. Control group at the same time point; # *p* <0.05 *vs*. Norepinephrine group at the same time point.

**Fig 3:**
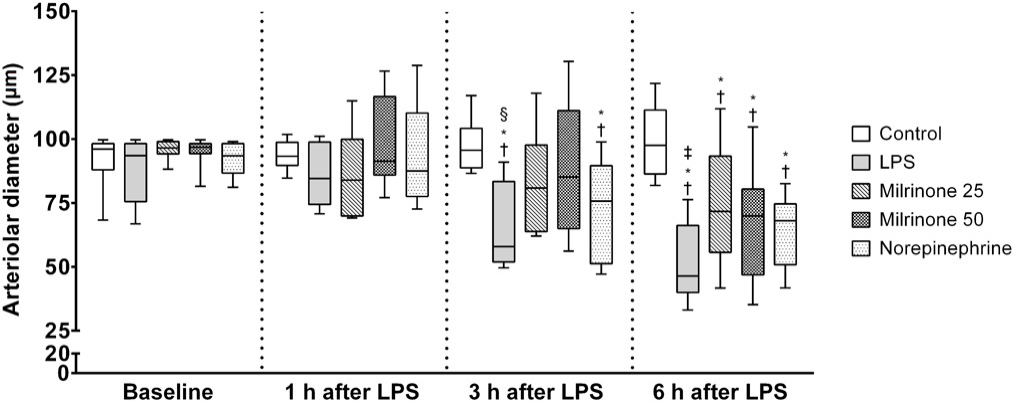
Evolution of arteriolar mean internal diameter during the experimental period. Sequential measurements were performed after one, three, and six hours of LPS injection. Control, non-endotoxemic animals (n = 10); LPS, endotoxemic animals (n = 10); Milrinone 25, endotoxemic and milrinone 0.25 μg.kg^-1^.min^-1^ treated animals (n = 10); Milrinone 50, endotoxemic and milrinone 0.50 μg.kg^-1^.min^-1^ treated animals (n = 10); Norepinephrine, endotoxemic and norepinephrine treated animals (n = 10). † *p* <0.05 *vs*. group baseline; * *p* <0.05 *vs*. Control group at the same time point; ‡ *p* <0.05 *vs*. Milrinone 25 group at the same time point; § *p* <0.05 *vs*. Milrinone 50 group at the same time point.

MAP and HR basal values were not significantly different among the experimental groups and were comparable to control values from healthy animals reported in the literature [17]. Systemic administration of LPS elicited similar reductions of MAP in all endotoxemic groups (Fig. 2—1 h after LPS). No abrupt decrease of MAP was observed shortly after the milrinone bolus. Additionally, no further reduction of MAP occurred after initiation of continuous milrinone infusion, so MAP remained comparable among LPS, Milrinone 25, and Milrinone 50 groups throughout the experimental period (Fig. 2). After initiation of drug infusions, a significantly higher MAP was observed in norepinephrine treated animals than in all other endotoxemic groups (Fig. 2—3 h and 6 h after LPS). HR was comparable among groups at each time point (Table 1).

**Table 1:** Heart rate and venular mean internal diameter evolution during the experimental period.

		**Control**	**LPS**	**Milrinone 25**	**Milrinone 50**	**Norepinephrine**
Heart rate (bpm)	Baseline	405.3±44.5	425.7±27.9	434.0±31.1	414.9±26.8	403.2±39.9
	1 hour after LPS	407.6±49.1	432.3±39.0	418.7±42.0	423.9±28.7	413.5±35.8
	3 hours after LPS	403.2±54.6	415.3±50.1	418.4±40.6	403.5±48.2	390.5±40.9
	6 hours after LPS	398.0±53.2	397.4±55.6	413.3±41.4	417.9±57.3	378.3±46.1
Venular diameter (μm)	Baseline	85.5±13.0	78.4±14.6	83.5±11.7	84.2±11.5	80.4±10.0
	1 hour after LPS	91.1±10.3	85.1±11.0	90.1±8.9	91.8±17.4	91.4±17.6
	3 hours after LPS	86.0±9.3	90.5±11.9	95.9±8.5	95.5±19.0	88.0±19.3
	6 hours after LPS	89.1±14.0	84.5±9.8	90.1±11.2	80.3±18.7	88.1±23.5

Data are presented as means±SD for each group. Control, non-endotoxemic animals (n = 10); LPS, endotoxemic animals (n = 10); Milrinone 25, endotoxemic and milrinone 0.25 μg.kg^-1^.min^-1^ treated animals (n = 10); Milrinone 50, endotoxemic and milrinone 0.50 μg.kg^-1^.min^-1^ treated animals (n = 10); Norepinephrine, endotoxemic and norepinephrine treated animals (n = 10).

### Arteriolar and venular mean internal diameters

At baseline, there were no significant differences in arteriolar and venular mean internal diameters between study groups. Arteriolar mean internal diameter was significantly reduced by endotoxemia in LPS group when compared with baseline. After initiation of drug infusions, a wider arteriolar mean internal diameter was observed in milrinone treated animals than in both LPS and Norepinephrine groups (*p* <0.05 *vs*. LPS group; Fig. 3—3 h and 6 h after LPS). No significant differences were observed between LPS and Norepinephrine groups (Fig. 3). In terms of venular mean internal diameter, LPS elicited slight venodilatation but there were no significant differences between study groups at any time point (Table 1).

### Capillary perfusion (FCD and RBC-Vel)

**Fig 4:**
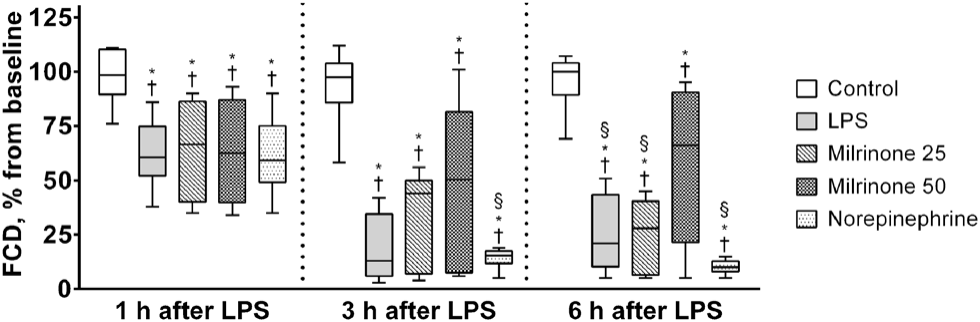
Evolution of functional capillary density (FCD) during the experimental period. Values of FCD are presented as ratios relative to baseline values. Sequential measurements were performed after one, three, and six hours of LPS injection. Control, non-endotoxemic animals (n = 10); LPS, endotoxemic animals (n = 10); Milrinone 25, endotoxemic and milrinone 0.25 μg.kg^-1^.min^-1^ treated animals (n = 10); Milrinone 50, endotoxemic and milrinone 0.50 μg.kg^-1^.min^-1^ treated animals (n = 10); Norepinephrine, endotoxemic and norepinephrine treated animals (n = 10). † *p* <0.05 *vs*. group baseline; * *p* <0.05 *vs*. Control group at the same time point; § *p* <0.05 *vs*. Milrinone 50 group at the same time point.

**Fig 5:**
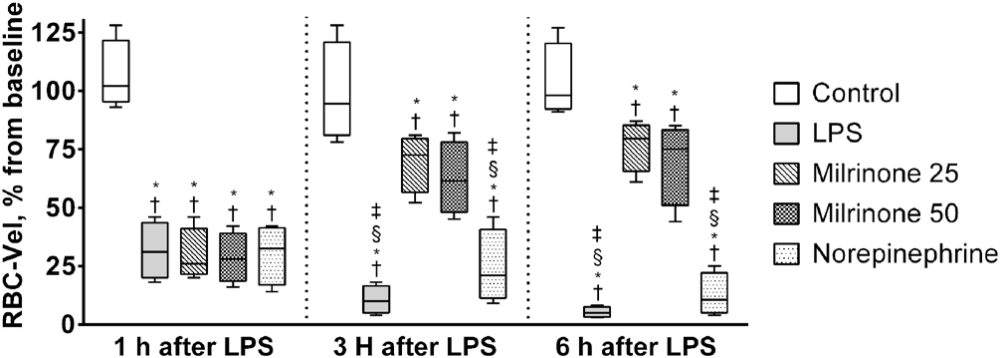
Evolution of red blood cell velocity in capillaries (RBC-Vel) during the experimental period. Values of RBC-Vel are presented as ratios relative to baseline values. Sequential measurements were performed after one, three, and six hours of LPS injection. Control, non-endotoxemic animals (n = 10); LPS, endotoxemic animals (n = 10); Milrinone 25, endotoxemic and milrinone 0.25 μg.kg^-1^.min^-1^ treated animals (n = 10); Milrinone 50, endotoxemic and milrinone 0.50 μg.kg^-1^.min^-1^ treated animals (n = 10); Norepinephrine, endotoxemic and norepinephrine treated animals (n = 10). † *p* <0.05 *vs*. group baseline; * *p* <0.05 *vs*. Control group at the same time point; ‡ *p* <0.05 *vs*. Milrinone 25 group at the same time point; § *p* <0.05 *vs*. Milrinone 50 group at the same time point.

At baseline, FCD and RBC-Vel did not significantly differ between study groups. LPS administration markedly decreased FCD and RBC-Vel (Figs. 4 and 5). Treatment with milrinone 0.50 μg.kg^-1^.min^-1^ (Milrinone 50 group) significantly attenuated the fall of both capillary perfusion parameters (*p* <0.05 *vs*. all other endotoxemic groups after 6 hours of LPS administration—Figs. 4 and 5) while treatment with milrinone 0.25 μg.kg^-1^.min^-1^ (Milrinone 25 group) significantly attenuated only the fall of RBC-Vel (*p* <0.05 *vs*. both LPS and Norepinephrine groups after 3 and 6 hours of LPS administration—Figs. 4 and 5). No significant differences were observed between LPS and Norepinephrine groups, although a trend towards lower FCD and higher RBC-Vel has been observed in norepinephrine treated animals (Figs. 4 and 5).

### Correlations between functional capillary density and mean arterial pressure

**Fig 6:**
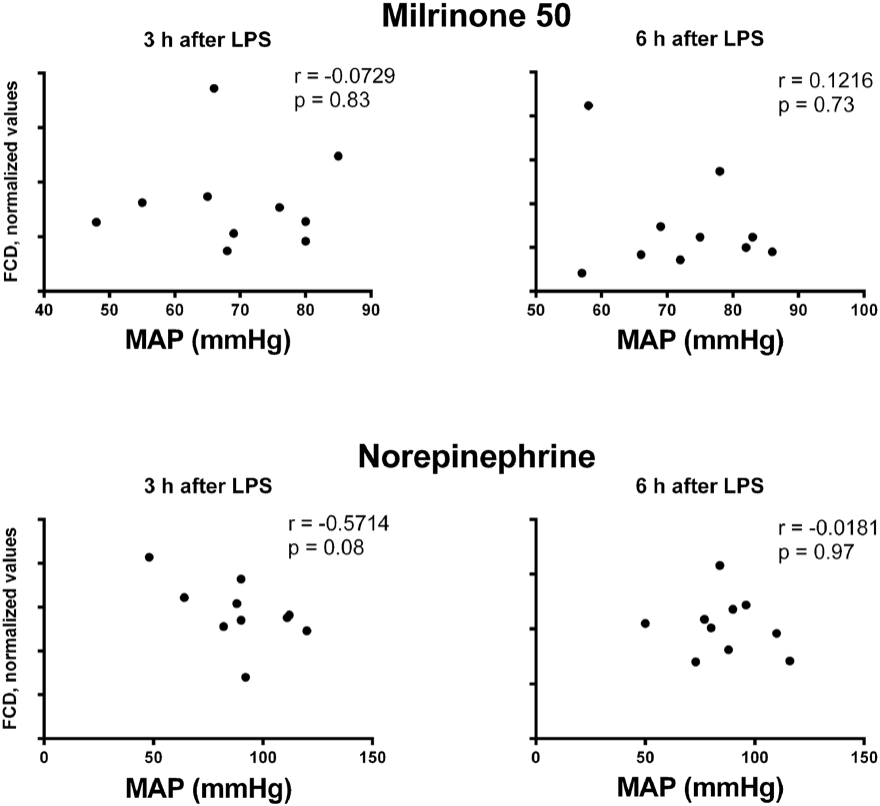
Scatter plots of correlations between functional capillary density (FCD) and mean arterial pressure (MAP). Spearman correlation coefficient and *p* values are shown for each correlation. n = 10 per time point and per group.

Regardless of the studied group (Milrinone 50 or Norepinephrine) or the analyzed time point (3 hours or 6 hours after LPS administration), FCD did not correlate to MAP (Fig. 6).

### Biochemical and hematological parameters

Biochemical and hematological parameters are presented on Table 2. Arterial lactate concentration was significantly higher in LPS, Milrinone 25, and Norepinephrine groups than in Control group; there were no statistical differences between Milrinone 50 and Control groups. Arterial pH and HCO_3_ were lower in LPS, Milrinone 25, and Norepinephrine groups compared with the Control group; there were no statistical differences between Milrinone 50 and Control groups. Leukometry and blood glucose were significantly lower in both LPS and Norepinephrine groups than in Control group; there were no statistical differences between Control and both milrinone treated groups. Hematocrit was significantly higher in both LPS and Norepinephrine groups than in Control group; there were no statistical differences between Control and both milrinone treated groups.

**Table 2:** Biochemical and hematological parameters.

	**Control**	**LPS**	**Milrinone 25**	**Milrinone 50**	**Norepinephrine**
Arterial lactate (mmol.l^-1^)	1.70±0.45	4.83±2.87*	3.31±1.30*	3.09±0.81	3.75±0.55*
pH	7.36±0.02	7.27±0.04*	7.27±0.05*	7.29±0.03	7.22±0.07*
Bicarbonate (mmol.l^-1^)	30.75±1.89	21.50±1.00*	26.95±1.55	28.00±2.00	22.22±1.98*
Hematocrit (%)	44.00±2.16	55.57±1.67*	51.25±2.75	51.50±2.88	56.60±7.43*
Blood Glucose (mg.dl^-1^)	68.67±18.87	35.56±7.60*	46.75±4.92	49.75±4.99	34.22±7.61*
Leukometry (n/mm^3^)	7,425±750	1,535±484*	2,475±450	3,055±918	2,489±919*

Blood sampling for biochemical and hematological analysis was performed in all groups at the end of the study period (6 h after LPS). Data are presented as means±SD for each group. Control, non-endotoxemic animals (n = 10); LPS, endotoxemic animals (n = 10); Milrinone 25, endotoxemic and milrinone 0.25 μg.kg^-1^.min^-1^ treated animals (n = 10); Milrinone 50, endotoxemic and milrinone 0.50 μg.kg^-1^.min^-1^ treated animals (n = 10); Norepinephrine, endotoxemic and norepinephrine treated animals (n = 10).

* p <0.05 vs. Control group.

### Seven days survival

**Fig 7:**
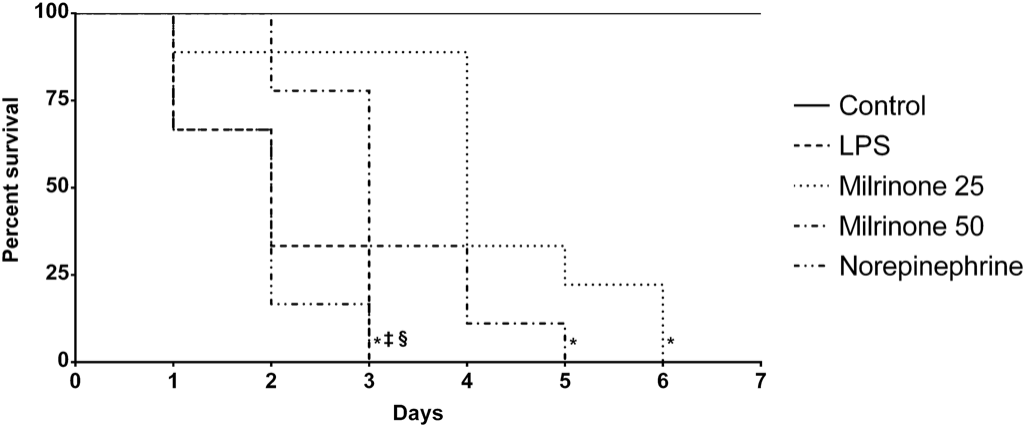
Kaplan-Meier survival curves. Control, non-endotoxemic animals (n = 10); LPS, endotoxemic animals (n = 10); Milrinone 25, endotoxemic and milrinone 0.25 μg.kg^-1^.min^-1^ treated animals (n = 10); Milrinone 50, endotoxemic and milrinone 0.50 μg.kg^-1^.min^-1^ treated animals (n = 10); Norepinephrine, endotoxemic and norepinephrine treated animals (n = 10). * *p* <0.05 *vs*. Control group; ‡ *p* <0.05 *vs*. Milrinone 25 group; § *p* <0.05 *vs*. Milrinone 50 group.

Median survival time was 2, 4, 3, and 2 for LPS, Milrinone 25, Milrinone 50, and Norepinephrine groups, respectively (*p* <0.05 for Control *vs*. endotoxemic groups; *p* <0.05 for LPS *vs*. milrinone treated groups; *p* <0.05 for Norepinephrine *vs*. milrinone treated groups; Fig. 7).

## Discussion

Microcirculatory dysfunction is one of the pathophysiological hallmarks of sepsis [18]. During this syndrome, the microcirculation can be recruited by reducing pathological shunting, promoting microcirculatory perfusion, supporting pump function, and controlling hemorheology and coagulation [18]. Milrinone, which acts on multiple of these mechanisms, may play a positive role in rescuing the microcirculation in sepsis. In fact, in the present study, milrinone proved to be beneficial in a validated endotoxemia rodent model that allows direct *in vivo* assessment of microcirculatory hemodynamics and perfusion dysfunction.

In regard to the model, the endotoxin dose used in our study was adjusted to affect microcirculatory parameters without the induction of severe hypotension simulating the hyperdynamic phase of sepsis (after initial resuscitation) [17]. This allowed the assessment of milrinone effects in a hemodynamic state different than that in which it is usually used. In Milrinone 50 group, milrinone was administered at a dose rate similar to that clinically given for cardiac output maintenance (bolus dose of 50 μg.kg^-1^ followed by a continuous IV infusion of 0.5 μg.kg^-1^.min^-1^). In previous experimental study by Kume et al. [12], at this dose, milrinone demonstrated beneficial microcirculatory and anti-inflammatory effects in a rodent model of hepatic ischemia/reperfusion injury without influence on animals’ macrohemodynamic parameters. As some effects of milrinone are dose-dependent, we have also chosen to test a treatment regimen with half of that dose (Milrinone 25 group). Norepinephrine was administered at a dose rate similar to that clinically given for septic patients.

Although milrinone is largely used for cardiac support, it produces anti-inflammatory effects independent of its inotropic or vasodilatatory activity. Cyclic adenosine monophosphate (cAMP) signal transduction has an important role in the inflammatory pathway [12]. It has already been shown that milrinone suppresses the expression of cytokines, such as interleukin 6 (IL-6), tumor necrosis factor-α (TNF-α), monocyte chemoattractant protein-1 (MCP-1), and macrophage inflammatory protein-2 (MIP-2), by increasing cAMP levels [19–21]. Furthermore, milrinone could modulate the response to cytokines as it decreases the expression of nuclear factor-κB (NF-kB) [21]. Anti-inflammatory effects of milrinone were further demonstrated when this drug attenuated the elevation of serum amyloid A values (an acute phase marker) after cardiopulmonary bypass [13]. Unfortunately, in our study we were unable to quantitate these inflammation-related molecules because there is no standardized laboratory test for golden Syrian hamsters. Failing to quantitate plasma markers of inflammation, we have chosen to investigate the inflammatory response through evaluation of surrogate markers: leukometry, blood glucose, and hematocrit.

Since endotoxemia is a known up-regulator of leukocyte-endothelial interactions, the observed leukometry differences after endotoxemia induction are likely to be related to activation and emigration of neutrophils out of the microvascular bed and to an accumulation of leukocytes in organs, such as lung and liver (leukocyte sequestration). This leukopenia becomes obvious 30 minutes after LPS infusion and usually persists for 8 hours after a single dose [17]. As tissue and organ accumulation of leukocytes are triggered and amplified by pro-inflammatory mediators, it may be suggested that the resultant leukopenia could be a marker of the inflammatory process that leads to microvascular dysfunction. In our study, milrinone treated animals showed greater leukocyte count, supporting its anti-inflammatory effect. Corroborating our findings, Jung et al. [21] have shown that milrinone decreases intercellular adhesion molecule-1 (ICAM-1), a key adhesion molecule involved with firm adhesion of leukocytes to the endothelium, which is one of the crucial steps of neutrophils emigration [22]. Moreover, Kume et al. [12] have demonstrated that milrinone treatment results in significant reduction of leukocyte-endothelial interactions (rolling and adherence), decreasing diapedesis. Once migration has occurred, tissue damage happens as a consequence of the release of an array of inflammatory mediators, cytotoxic enzymes, and oxygen radicals. So, generalized activation and sequestration of neutrophils are likely to contribute to the widespread microvascular injury and subsequent endothelial damage observed in sepsis, which represents a central step in the development and progression of multiple organ failure [18, 23]. In this way, the observed anti-inflammatory effects of milrinone could be beneficial to the microcirculation.

The observed development of hypoglycemia after LPS administration is a common finding during inflammatory states in small animals and anti-inflammatory drugs could mitigate this hypoglycemic response [24]. Thus, the relative higher glucose level found in milrinone treated hamsters may be related to lower inflammatory response. Unfortunately, our blood glucose results may be biased by stimulating c-AMP effects on glycogenolysis, lipolysis, and gluconeogenesis that may elicit an elevation of glycemia independently of the inflammatory status [12]. Finally, hematocrit increasing after endotoxemia induction, suggesting increased capillary leakage, was not observed in milrinone treated animals. This hematological parameter has been used as an indirect marker of plasma leakage in the context of endothelial-damaging infectious diseases like dengue virus infection [25]. The absence of hemoconcentration in milrinone groups may be related to anti-inflammatory effects of the drug and/or to protective effects on vascular endothelial barrier function as it has already been shown that a cAMP-dependent protein kinase prevents increased endothelial permeability induced by inflammatory mediators [26].

Milrinone treatment attenuated arteriolar LPS-induced vasoconstriction. This behavior is probably associated with the vasodilatatory effects of the drug. As a cAMP-elevating vasodilator, milrinone acts in the vascular smooth muscle cell by reducing muscle sensitivity to calcium influx [27]. An endothelium-dependent mechanism linked to endothelial nitric oxide-cyclic guanosine monophosphate (eNO-cGMP) signal cascade may have a role in milrinone-induced vasodilatatory response [12, 28].

Analyzing RBC-Vel temporal evolution, our study showed that administration of both milrinone dose regimens was associated with significant attenuation of RBC-Vel reduction induced by LPS administration. This may be, at least in part, explained by the positive inotropic activity of the drug, improving cardiac output. With a similar assumption, we can explain the observed trend towards better RBC-Vel found with the use of norepinephrine (a drug with beta-1 adrenergic activity) in comparison to the LPS group.

When FCD temporal evolution was considered, our study showed that only the full dose of milrinone (0.50 μg.kg^-1^.min^-1^) was associated with significant attenuation of LPS-induced decrease of FCD. Several factors are related to the microcirculatory impairment observed after endotoxemia induction, such as vasoconstriction, increased leukocyte-endothelium interactions, and platelet/fibrin clot formation [4]. Since FCD differences between both milrinone groups cannot be explained by changes in arteriolar mean internal diameter, macro-hemodynamic changes, or anti-inflammatory effects, we may speculate that differences in antithrombotic activity between the two groups may have been crucial to the observed differences since capillary obstruction by microthrombi may hamper adequate capillary flow [29, 30]. Although milrinone is a known impairer of platelet activation, its antithrombotic activity is dose-dependent, thereby increased blood concentrations of milrinone are associated with increased inhibition of adenosine diphosphate and arachidonic acid-induced platelet activation [14]. The reduction of microvascular platelet aggregation facilitates capillary blood flow, improving tissue perfusion [31].

Lower values of pH and HCO_3_ found in LPS, Milrinone 25, and Norepinephrine groups compared with Control group are indicative of metabolic acidosis, probably secondary to tissue hypoperfusion. In these groups, the presence of metabolic acidosis associated with hyperlactatemia denotes a high risk clinical condition [32, 33]. On the other hand, lower arterial lactate concentrations observed in Milrinone 50 group suggests that this group had, at the moment of blood sampling, better tissue perfusion compared with all other endotoxemic groups corroborating our microcirculatory findings.

Milrinone treated animals had increased survival compared with animals in both LPS and Norepinephrine groups. Because LPS-induced mortality is mainly dependent on activation of the inflammatory cascade, it is understandable that a substance with anti-inflammatory properties (milrinone) could improve survival.

Clinical use of milrinone for sepsis treatment has been held back by fear of induction of severe hypotension. Despite theoretical concerns, no hemodynamic worsening was observed in our and other experimental studies [11, 12] suggesting that this should not be considered an impediment for its use in septic patients. It is argued that the augmentation of cardiac output (inotropic effect) compensates the decreased systemic vascular resistance (vasodilatatory effect) preserving MAP. Even if MAP decreases during milrinone infusion, there is growing body of evidence indicating that arterial blood pressure is not a direct determinant of either microvascular perfusion or sepsis survival [34, 35]. Many authors have already demonstrated limited correlation between systemic hemodynamic parameters and microcirculatory function in such a way that the recovery of macrohemodynamic stability does not necessarily match with microhemodynamic improvement [36, 37]. In line with these findings, no correlation between FCD and MAP was found in our study, no matter which drug was infused (milrinone 0.50 μg.kg^-1^.min^-1^ or norepinephrine). Although it seems paradoxical, the use of a vasodilator agent in a disease with low systemic vascular resistance may even improve microvascular perfusion. This was true for milrinone (0.50 μg.kg^-1^.min^-1^) when it markedly attenuated LPS-induced arteriolar vasoconstriction and FCD decrease. Because sepsis is characterized by irregular distribution of flow with pathological shunting due to disproportionate microvascular vasoconstriction (secondary to disturbance of vasomotor auto-regulation), it is understandable why a vasodilator could recruit failing microcirculatory units ameliorating tissue perfusion [36, 38]. There are many other mechanisms involved in microcirculatory dysfunction during sepsis, such as the presence of microthrombi, capillary leakage, and leukocyte rolling and adherence [4, 36]. Milrinone has pleiotropic microcirculatory effects aside from its vasodilatatory actions and could act against many of these mechanisms having greater therapeutic potential than compounds directed against a single target [39].

Current guidelines [7] recommend norepinephrine as the first-line vasopressor for patients in persistent septic shock despite fluid resuscitation, so we considered this drug for control treatment in our experiments. This catecholamine is usually used to increase MAP above the auto-regulatory threshold (65 mmHg) as an effort to increase perfusion pressure [40, 41]. Nevertheless, Dubin et al. [41] have demonstrated that increasing arterial blood pressure with norepinephrine does not improve microcirculatory blood flow. In fact, norepinephrine not only does not recruit the microcirculation as it actually reduces microcirculatory perfusion, even though arterial blood pressure is restored [41–44]. This is in line with our findings in which norepinephrine treated animals showed the worst FCD values but the best MAP levels among all endotoxemic groups. Besides its detrimental microcirculatory effects, norepinephrine showed no anti-inflammatory effects, as depicted by surrogate markers of inflammation. Actually, some authors argue that norepinephrine has pro-inflammatory effects, which would be an extremely inappropriate characteristic for a sepsis treatment [45]. Considering all these characteristics, we might question norepinephrine’s primacy as the vasoactive drug of choice in sepsis.

We are aware that our study has some limitations. First, although we know that fluid therapy is recommended on early management of severe sepsis and septic shock, our animals were not fluid resuscitated because this study was designed to evaluate the effects of milrinone on the microcirculation independently of fluid therapy effects. Second, LPS injection model has some disadvantages because it is acute in nature and may not reproduce many of the clinical features of sepsis syndrome. Indeed, some authors do not consider it a model of sepsis, but rather a model of endotoxemia, so we cannot generalize/translate our results to the much more complex clinical scenario of human sepsis [46, 47]. On the other hand, it is a well-established and validated experimental model that offers advantages over other models of sepsis, since experiments are easy to perform and its dosage can be controlled and adjusted to the actual body weight of the animal resulting in a predictable insult. Another advantage of this model is that homogeneity and reproducibility of microvascular responses are consistently obtained [17]. Third, we recognize that the study of the skin and subcutaneous muscle microcirculation may not be representative of microcirculatory changes in splanchnic organs. Given the crucial importance of splanchnic perfusion in the pathophysiology of sepsis, this could be considered a limitation of the microcirculatory model used in our study. However, the first reactions after endotoxin administration seem to be comparable in different tissues and organs [17]. The hamster skinfold window chamber model developed by Endrich and co-workers [15] is widely used for microvascular studies in unanesthetized animals [23, 29, 48–51]. Unlike acute microcirculatory models, this model permits the existence of a recovery period between the surgical manipulation for chamber implantation and the actual experiments. This period allows the recovery of the microcirculatory function affected by surgical trauma. Furthermore, compared with other microcirculatory models, skinfold chamber experiments can be performed without induction of general anesthesia, which has hemodynamic, immune, and microvascular effects of its own. The animals tolerate the chamber and catheters well and show no signs of discomfort, as indicated by normal daily feeding, cleaning, and sleeping habits [52]. Thus, administration of sedatives and/or analgesics is not formally required. Fourth, although indirect parameters, such as blood gas analyses and improved survival in milrinone-treated groups, suggest that milrinone‘s beneficial effects on microcirculation are effective to protect organs from failure, we did not conduct histopathological or specific biochemical evaluations, limiting our conclusions regarding end-organ function. Finally, the evaluation of surrogate markers of inflammation and vascular endothelial barrier function rather than more direct parameters, such as cytokines and VE-cadherin/claudin-5, is a species-specific methodological limitation of our study.

## Conclusions

In our study, milrinone (0.50 μg.kg^-1^.min^-1^) markedly attenuated LPS-induced arteriolar vasoconstriction and capillary perfusion deficits (RBC-Vel and FCD decrease) suggesting that it yields a protective effect on endotoxemic animals’ microcirculation. Interestingly, these beneficial effects were achieved using an endotoxemia model that simulates the hyperdynamic phase of sepsis, a commonly found hemodynamic state in adult septic patients. Furthermore, milrinone treatment during endotoxemia showed anti-inflammatory properties, attenuating changes in surrogate markers of inflammation, and improved survival. Norepinephrine did not recruit the microcirculation nor demonstrated anti-inflammatory effects. Further studies in experimental models closer to human sepsis are required to confirm these results.
